# Differential impact of high-salt levels in vitro and in vivo on macrophage core functions

**DOI:** 10.1007/s11033-024-09295-x

**Published:** 2024-02-24

**Authors:** Linda Müller, Aya Rafea Nasr, Bettina Jux, Nikola Makdissi, Justin Wayne Trowbridge, Susanne V. Schmidt, Joachim L. Schultze, Thomas Quast, Jonas Schulte-Schrepping, Waldemar Kolanus, Elvira Mass

**Affiliations:** 1 Molecular Immunology and Cell Biology, Life & Medical Sciences (LIMES) Institute, University of Bonn, 53115 Bonn, Germany; 2 Developmental Biology of the Immune System, Life & Medical Sciences (LIMES) Institute, University of Bonn, 53115 Bonn, Germany; 3 Genomics & Immunoregulation, Life & Medical Sciences (LIMES) Institute, University of Bonn, Bonn, Germany; 4 Systems Medicine, Deutsches Zentrum Für Neurodegenerative Erkrankungen (DZNE) E.V, Bonn, Germany; 5 PRECISE Platform for Single Cell Genomics and Epigenomics, DZNE and University of Bonn, Bonn, Germany

**Keywords:** Macrophage, Salt sensing, Inflammation, Phagocytosis, Efferocytosis, High-salt diet

## Abstract

The consumption of processed food is on the rise leading to huge intake of excess dietary salt, which strongly correlates with development of hypertension, often leading to cardiovascular diseases such as stroke and heart attack, as well as activation of the immune system. The effect of salt on macrophages is especially interesting as they are able to sense high sodium levels in tissues leading to transcriptional changes. In the skin, macrophages were shown to influence lymphatic vessel growth which, in turn, enables the transport of excess salt and thereby prevents the development of high blood pressure. Furthermore, salt storage in the skin has been linked to the onset of pro-inflammatory effector functions of macrophages in pathogen defence. However, there is only little known about the mechanisms which are involved in changing macrophage function to salt exposure. Here, we characterize the response of macrophages to excess salt both in vitro and in vivo. Our results validate and strengthen the notion that macrophages exhibit chemotactic migration in response to salt gradients in vitro. Furthermore, we demonstrate a reduction in phagocytosis and efferocytosis following acute salt challenge in vitro. While acute exposure to a high-salt diet in vivo has a less pronounced impact on macrophage core functions such as phagocytosis, our data indicate that prolonged salt challenge may exert a distinct effect on the function of macrophages. These findings suggest a potential role for excessive salt sensing by macrophages in the manifestation of diseases related to high-salt diets and explicitly highlight the need for in vivo work to decipher the physiologically relevant impact of excess salt on tissue and cell function.

## Introduction

Salt, an essential component of the human diet, plays a pivotal role in maintaining various physiological processes, including osmoregulation, nerve conduction, and muscle function. Sodium chloride (NaCl), the most common dietary salt, has been a subject of substantial scientific interest due to its impact beyond electrolyte balance. Historically, the role of NaCl in immunity was primarily associated with osmotic balance and cell homeostasis. However, recent investigations have unveiled its intricate involvement in immunomodulation. These studies have shed light on the complex interplay between dietary salt intake and immune system modulation, particularly in the context of macrophage functions [1]. Macrophages, as key components of the innate immune system, serve as sentinels in the body’s defense against pathogens, tissue repair, and immune regulation. Their plasticity and functional diversity enable them to perform a spectrum of roles ranging from pro-inflammatory and anti-microbial responses, to tissue-repairing and anti-inflammatory activities [2, 3]. Emerging evidence suggests that the microenvironment, including the concentration of NaCl, critically influences macrophage behavior and functionality [4–6]. High salt concentrations have been demonstrated to exert profound effects on macrophage phenotypes, altering, e.g., their polarization status and consequently impacting immune responses and trained immunity [7–10]. Salt levels have also been linked to changes in macrophage metabolic pathways, including glycolysis and oxidative phosphorylation. An altered cellular metabolism significantly impacts macrophage functions, affecting their phagocytic capacity, antigen presentation, and overall immune response [11–16]. Understanding the intricate relationship between dietary salt and macrophage functions is of paramount importance in elucidating the mechanisms underlying immune system modulation and disease pathogenesis. In this paper, we attempt a systematic analysis of macrophage core functions, such as phagocytosis and motility, and provide a juxtaposition of various experimental protocols, including short-term exposure of macrophages to excess NaCl in vitro and ex vivo. Importantly, we demonstrate here altered macrophage core functions which have been re-programmed by long-term in vivo exposure to high dietary salt.

## Materials and methods

### Cell culture

The murine macrophage cell line J774A.1 mCherry was purchased from ATCC and modified by René Neuhaus from Pallasch Lab (Cologne, Germany) using a mCherry MLS empty vector. The human-MYC/BCL2 (hMB) GFP^+^ cell line (strain 102) was generated by Leskov et al. [17]. Cells were cultured in Dulbeccos modified Eagels Medium (DMEM) (Cat.No. P04-03550, PAN-Biotech) supplemented with 10% Fetal Bovine Serum (Sigma) and 1% Penicillin-Streptavidin (Biochrom) in a 5% CO_2_ atmosphere with 95% humidity at 37 °C. Bone marrow-derived macrophages (BMDMs) and peritoneal macrophages for in vitro experiments were isolated from C57BL/6JRcc mice. For differentiation into BMDMs, bone marrow-derived cells were put on 10 cm dishes with DMEM treated with 10 ng/ml recombinant murine M-CSF (Peprotech) for 7 days. Peritoneal macrophages were isolated via peritoneal lavage by injecting the peritoneum with 5 ml cold PBS, shaking the mouse for 2 min, then aspirating the injected fluid. Peritoneal lavage was centrifuged 5 min at 400 g, supernatant was discarded and the cell pellet was resuspended in DMEM for culturing. After culturing them for 2 h at 37 °C, the non-adherent cells were removed and adherend cells were supplemented with new media.

### In vitro migration assay

Chemotaxis of the macrophage cell line J774A.1 and BMDMs was analysed using a modified Boyden chamber (transwell) assay with 8 µm pore-size membrane inserts (Corning™ #3422, Fisher Scientific). 2 × 10^5^ cells were suspended in serum-reduced (0.5% FCS) DMEM and put in the upper well 1 h in advance in order to adhere to the membrane. Afterward, the lower chamber was filled with DMEM (standard medium has 109.5 mM NaCl) containing 10 nM C5a, additional NaCl (Merck) with concentrations ranging from 30 to 70 mM and 120 mM Mannitol as osmolarity control. After 20 h, medium and leftover cells on top of the membrane were removed using cotton swabs, and transmigrated cells on the bottom of the membrane were fixed with 4% PFA and stained with 5 µM Vybrant CFDA-SE in PBS (Invitrogen). For each insert, 5 random fields were observed using an inverted Nikon Eclipse TE 200-E fluorescence microscope (Nikon), equipped with a PlanFluor DL 10x/0.30 N.A. objective (Nikon). The number of transmigrated cells was analyzed by the counting tool in Fiji (ImageJ).

### Flow cytometry

BMDMs were differentiated as described above and treated either with control, 60 mM excess salt or 120 mM mannitol for 24 h. Afterwards the cells were stained with an antibody mix (BD Biosciences) containing Tim4 BUV-563 (clone RMT4-54), CD16.2 BV785 (9E9), CD64 PE Dazzle (X54-5/7.1), MerTK PE-Cy7 (2B10C42), CD32b APC (AT130-2). The surface marker expression was measured using BDSymphony A5 (BD Biosciences) and analyzed with FlowJo analysis software (version 10.9.0).

### Phagocytosis assay

Phagocytosis of BMDMs and peritoneal macrophages was analyzed using fluorescent 500 nm polystyrene beads (PolySciences). For in vitro experiments, cells were put on 12-well plates at a concentration of 1 × 10^5^/ml in DMEM. After cells adhered to the plate, the supernatant was aspirated and replaced by a fresh DMEM medium containing the beads, different excess NaCl concentrations ranging between 26 and 104 mM or the respective osmolarity control (104 mM Mannitol). For ex vivo experiments, BMDMs and peritoneal macrophages were derived from C57BL/6JRcc mice which were on their respective diet for one week or three months. Cells were put on 12-well plates without adding excess salt. Fluorescent polystyrene beads were added for 5 h, cells were stained with CD11b APC (clone: M1/70) and F4/80 APC (BM8) antibodies (BioLegend) and measured using a flow cytometer (BD LSRII, BD Biosciences). Phagocytosed beads were analyzed using the FlowJo analysis software (version 10.9.0).

### Efferocytosis assay

Efferocytosis of BMDMs and peritoneal macrophages was analyzed by two different approaches using two types of apoptotic cells. For the first approach, GFP^+^ hMB lymphoma B cells were put on a heat shock (42 °C) for 90 s to induce apoptosis, followed by resuspension in the respective treatment (DMEM with excess NaCl or Mannitol). In the second approach, thymus cells were used as apoptotic cells. For cell isolation, the thymus of 4–5-week-old C57BL/6JRcc mice was harvested, plunged through a 100 µm strainer, washed with RPMI (Cat. No. P04-03550, PAN-Biotech), centrifuged and resuspended in DMEM to a concentration of 20 × 10^6^/ml, and plated in 6-well plates. For inducing apoptosis, thymus cells were treated with 5 µM Dexamethasone per well for 5 h. Next, they were stained using Amine-reactive pHrodo™ Red dye (Thermofisher). Cells were added to 12-well plates with pre-plated 1 × 10^5^ macrophages per well in DMEM in a ratio of 1:10. After 24 h (hMBs) or 2 h (thymus cells), apoptotic cells were removed, macrophages were stained with CD11b and F4/80 APC antibodies (BD Biosciences) and measured using a flow cytometer (FACS LSR II, BD Biosciences). The efferocytosis rate was analyzed using the FlowJo analysis software. For the ex vivo condition, BMDMs and peritoneal macrophages were derived from C57BL/6JRcc mice which were on NSD or HSD for one week or three months. For the in vitro condition, excess salt and osmolarity controls were added to the cells for 24 h.

### Annexin V/Propidium Iodide staining

For apoptosis assessment the Annexin V Apoptosis Detection kit with Propidium Iodide (PI) (Biolegend) was used according to the manufacturer’s instructions and BMDMs were measured using a LSR II flow cytometer. First, single cells were gated. PI + cells were identified as dead cells, Annexin V + cells were identified as apoptotic cells. The remaining cells were identified as live cells.

### Animal work

All animal experiments were conducted in a licensed animal facility following the German law on the protection of experimental animals and were approved by local authorities of the state of Nordrhein-Westfalen (Landesamt für Natur, Umwelt und Verbraucherschutz NRW, license number 81–02.04.2021.A203). Mice were sacrificed either via CO_2_ inhalation (in vitro experiments) or by transcardial perfusion (in vivo experiments). Diets were purchased from ssniff (control diet (CD): E15748-047, high-salt diet (HSD) #E15431-34). For CD, mice received tap water, for HSD conditions, the water contained 0.9% NaCl.

### Bulk RNA sequencing and analysis

Bulk RNA sequencing was performed using BMDMs. Bone marrow was isolated from *Cx3cr1*^*GFP*^ mice (C57BL/6 J background, Jackson #000664). 10 × 10^6^ cells were cultured in VLE-RPMI 1640 medium (Art.No. F 1415, Biochrom) containing 10% FCS (Sigma), 1% Pen/Strep (PAA), and 10 ng/ml M-CSF (Peprotech). After seven days of culturing, half of the supernatant including the non-adherent cells was discarded and fresh medium was added. Furthermore, the cells were treated with an additional 62 mM NaCl for 24 h. Non-adherent cells were discarded and adherent cells were detached by application of 2 mM EDTA in PBS and used for RNA sequencing. For RNA isolation, cells were lyzed in Qiazol (Qiagen) and total RNA was extracted with the miRNeasy kit (Qiagen) according to the manufacturer’s protocol. Subsequently, polyadenylated RNA was purified from the total RNA using Oligo-dT-attached magnetic beads. After fragmentation, the RNA was converted into libraries of double-stranded cDNA molecules with ligated adapter molecules using the Illumina TruSeq RNA Sample Preparation Kit v2 without preservation of strand information. Size selection and purification of cDNA fragments was conducted favouring 200 bp in length. After cluster generation on a cBot station, RNA sequencing was performed on a HiSeq1500 (Illumina) in a 75 bp single-end run. After base calling and de-multiplexing, quality control of the raw sequencing reads was performed using fastQC 0.11.9 (https://www.bioinformatics.babraham.ac.uk/projects/fastqc/) and summarized using MultiQC v1.14 (https://multiqc.info/). The 75 bp single-end reads were trimmed using fastp v0.20.0 and aligned to the murine reference genome mm10 vM32 from GENCODE using STAR v2.7.10b (https://github.com/alexdobin/STAR) [18] with –quantMode GeneCounts. Aligned reads sorted by coordinates were indexed using samtools v1.16.1. The alignment pipeline was written and executed using SnakeMake 7.20.0 (https://snakemake.github.io/) [19]. Downstream data analysis was performed in R version 4.3.1 mainly using the R package DESeq2 version 1.40.2 [20]. Raw counts were imported into R and a DESeq object was constructed using the DESeqDataSetFromMatrix function. Genes with less than a total count of 10 were excluded from the analysis resulting in 15,903 present genes. Estimation of size factors and dispersions was performed per gene using default parameters in DESeq2. Differential expression analysis was performed using DESeq2 comparing NaCl treated samples to controls. Gene ontology enrichment analysis in the sets of differentially expressed genes was performed using enrichGO function of the R package ClusterProfiler version 4.8.2 [21].

### Statistics

Experiments were performed with at least three biological replicates each including three technical replicates. Data are shown as mean ± SD. One-way ANOVA with Tukey’s post hoc test and unpaired Student’s *t*-test with values of *p* < 0.05 considered significant were performed using the GraphPad Prism software (v 10.1.1).

## Results and discussion

**Fig. 1:**
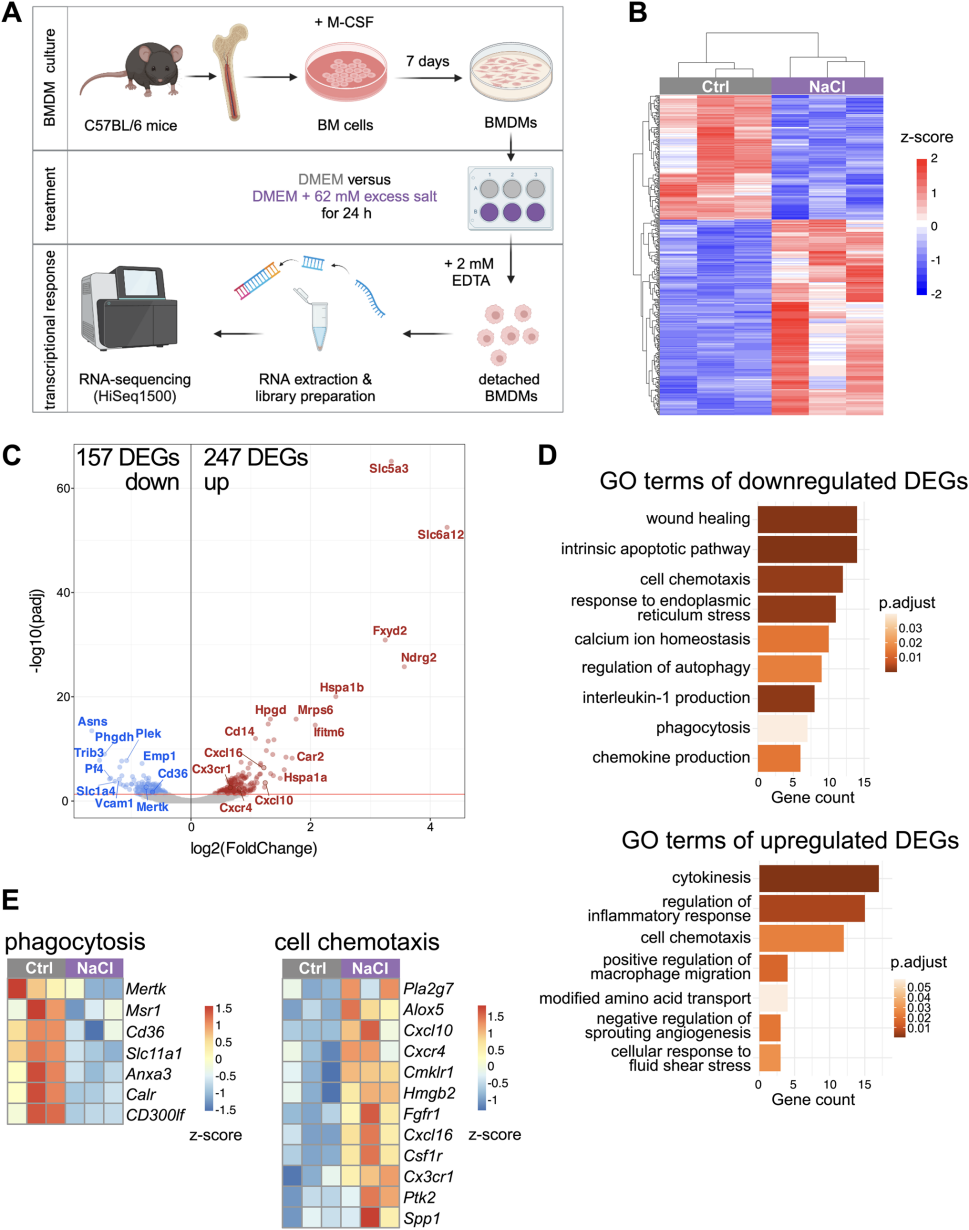
Transcriptional changes in bone-marrow derived macrophages (BMDMs) in response to sodium chloride (NaCl). **A** Experimental setup **B** Hierarchical clustering of all differentially expressed genes of unstimulated control cells (Ctrl) versus 62 mM excess NaCl samples. *n* = 3. **C** Volcano plot showing all expressed genes. Differentially expressed genes (DEG) are color-coded. **D** Gene Ontology (GO) terms of down- and upregulated DEG (*q*-value ≤ 0.01). **E** Relative expression of genes falling into the terms ‘phagocytosis’ and ‘cell chemotaxis’

To study the effects of an increased salt concentration on macrophage function, we first assessed transcriptional changes upon 62 mM excess NaCl treatment of bone-marrow-derived macrophages (BMDMs) after 24 h by performing bulk RNA-sequencing (Fig. 1A). Hierarchical clustering of all differentially expressed genes (DEG, 247 upregulated, 157 downregulated) revealed a clear response of BMDMs to high-salt conditions (Fig. 1B and C). Gene Ontology (GO) term enrichment analysis of DEG showed that genes falling into terms such as ‘cell chemotaxis’, ‘regulation of inflammatory response’, and ‘positive regulation of macrophage migration’ were upregulated, while downregulated genes fell into terms such as ‘wound healing’, ‘chemokine production’, ‘interleukin-1 production’, and ‘phagocytosis’ (Fig. 1D and E). Together, this data set indicates that macrophages sense excess salt in their environment and alter their transcriptional program, which may perturb their effector functions.

**Fig. 2:**
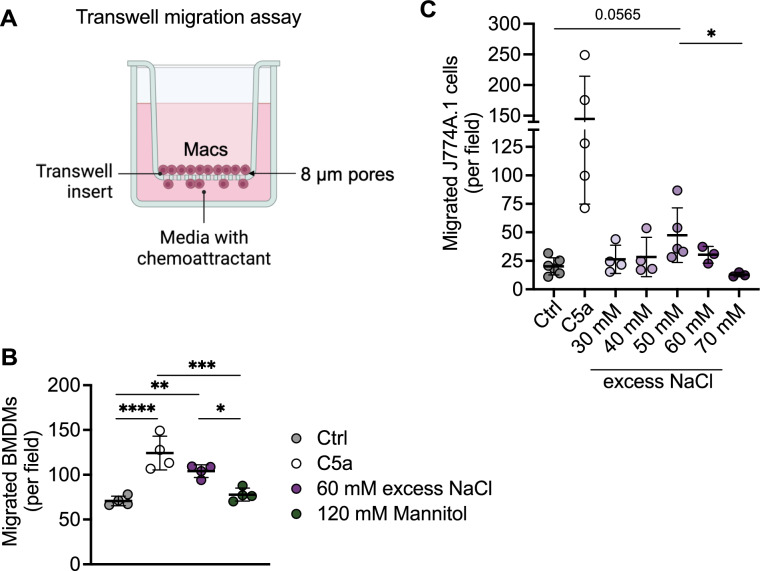
Migration behaviour of macrophages in response to sodium chloride (NaCl). **A** Experimental setup. **B** Transwell migration assay of bone-marrow derived macrophages (BMDMs) towards control DMEM medium (Ctrl), positive control (C5a), 60 mM excess NaCl and 120 mM mannitol. **C** Transwell migration assay of J774A.1 macrophages towards control DMEM medium (Ctrl), positive control (C5a), and different excess NaCl concentrations (30 mM – 70 mM). Significance was tested using one-way ANOVA with Tukey’s post hoc test. *p < 0.05, **p < 0.01, ***p < 0.001, ****p < 0.0001. For C, the positive control was not included to calculate significance. Each circle represents a biological replicate as the mean of 3 technical replicates. Shown is the mean ± SD

As BMDMs altered expression of genes related to cell chemotaxis and we have previously shown that macrophages can recognize NaCl as chemotactic stimulus [5], we validated that BMDMs migrate in the direction of excess salt concentration by using an in vitro transwell migration assay using 60 mM excess NaCl (Fig. 2A and B). 120 mM mannitol was used as osmolarity control. The macrophage chemoattractant complement component 5A (C5a) served as positive control. To assess the required concentration that can be sensed by macrophages in general, we performed transwell assays, using the immortalized murine J774A.1 macrophage cell line and increasing concentrations of NaCl. We observed that J774A.1 cells migrated mostly at 50 mM and 60 mM excess salt (Fig. 2C).

**Fig. 3:**
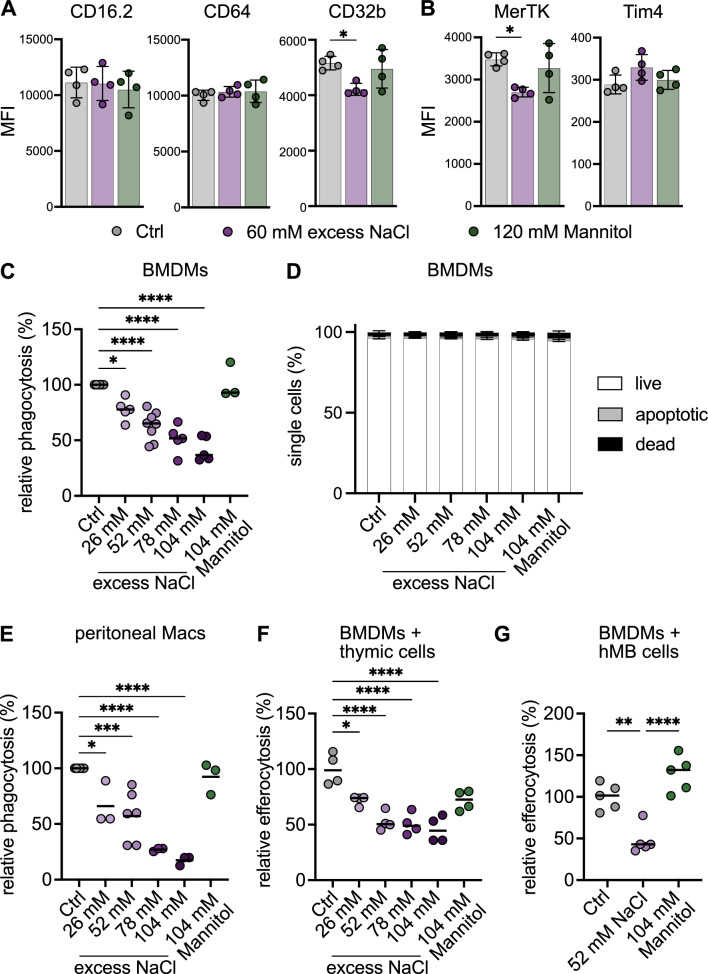
Phagocytosis and efferocytosis activity of macrophages in response to sodium chloride (NaCl) in vitro. **A** and **B** Flow cytometry analysis of phagocytosis (**A**) and efferocytosis (**B**) surface markers mean fluorescence intensity (MFI) expressed on bone-marrow derived macrophages (BMDMs) after 24 h culture with DMEM medium (Ctrl), 60 mM excess NaCl or 120 mM Mannitol. Error bars represent SD of the mean. **C** Phagocytosis of polystyrene beads by BMDMs after 24 h culture with DMEM medium (Ctrl), different excess NaCl concentrations (26 mM – 104 mM) or 104 mM mannitol. **D** Annexin V/PI assay with the same experimental setup as in (**C**). **E** Phagocytosis of polystyrene beads by peritoneal macrophages (Macs) with same experimental setup as in (C). **F** and **G** Efferocytosis of thymic cells (**E**) or hMB cells (**F**) by BMDMs using the same conditions as in (C-D). Significance was tested using one-way ANOVA with Tukey’s post hoc test. *p < 0.05, **p < 0.01, ***p < 0.001, ****p < 0.0001. Each circle represents a biological replicate as the mean of 3 technical replicates. For (D) *n* = 6 biological replicates. Shown is the mean ± SD

Phagocytosis and efferocytosis (phagocytosis of apoptotic cells) are core functions of macrophages, and were affected on a transcriptional level upon high-salt exposure (Fig. 1C). Therefore, we tested whether the abundance of phagocytosis-related Fc gamma receptors (FcγR) CD64, CD32b and CD16 were affected under high-salt conditions. Flow cytometry analyses revealed that the expression of inhibitory FcγR CD32b was downregulated by excess NaCl while CD64 and CD16 were not altered (Fig. 3A). Interestingly, the efferocytosis receptors MerTK and Tim4 showed an opposing behaviour with MerTK being downregulated, validating the transcriptional downregulation (Fig. 1D), and Tim4 being slightly upregulated upon NaCl treatment (Fig. 3B). Both receptors recognize phosphatidylserine on the surface of apoptotic cells and MertK, as part of the TAM receptor family, and Tim4 collaborate for efficient efferocytosis in distinct macrophage populations [22]. However, while Tim4 may enhance TAM-dependent efferocytosis, some macrophage populations act completely Tim4 independent for the recognition and uptake of apoptotic cells [22]. Therefore, depending on the expression pattern of phagocytosis and efferocytosis receptors on macrophage subpopulations in vivo, excess salt exposure may result in distinct perturbations of macrophage effector functions.

To test whether phagocytosis was affected upon excess salt, we treated BMDMs with different NaCl concentrations (26–104 mM) or 104 mM mannitol, which corresponds to the osmolarity reached by 52 mM excess NaCl, and added 500 nm polystyrene (PS) beads for 5 h. Uptake of fluorescently labelled PS beads by CD11b^+^F4/80^+^ BMDMs was assessed via flow cytometry indicating that increasing NaCl concentrations inhibit phagocytosis (Fig. 3C). Of note, cells did not undergo apoptosis due to increased NaCl concentrations (Fig. 3D). Similar results were observed using peritoneal macrophages (Fig. 3E). Further, we quantified BMDM efferocytosis of apoptotic thymocytes labelled with pHrodo, a pH-sensitive dye that fluoresces brightly in acidic environments, such as the phagosome. Also here, increasing NaCl concentrations inhibit efferocytosis, albeit a dose-dependent inhibition as observed in the phagocytosis assay is lacking (Fig. 3F). Interestingly, a plateau of efferocytosis inhibition to ~ 45% seems to be reached already at 52 mM excess salt representing the physiologically relevant salt concentration cells may experience under high-salt diet conditions [4]. Next, we assessed efferocytosis using the fluorescent tumour line hMB, where only 10–20% of cells were apoptotic, representing a more physiological abundance of apoptotic cells. Similarly to results obtained with apoptotic thymocytes, the relative efferocytosis rate at 52 mM excess salt was decreased to ~ 43% (Fig. 3G). In summary, in vitro results using BMDMs indicate that excess salt inhibits both phagocytosis and efferocytosis, possibly by regulating surface receptor expression important for these macrophage core functions.

**Fig. 4:**
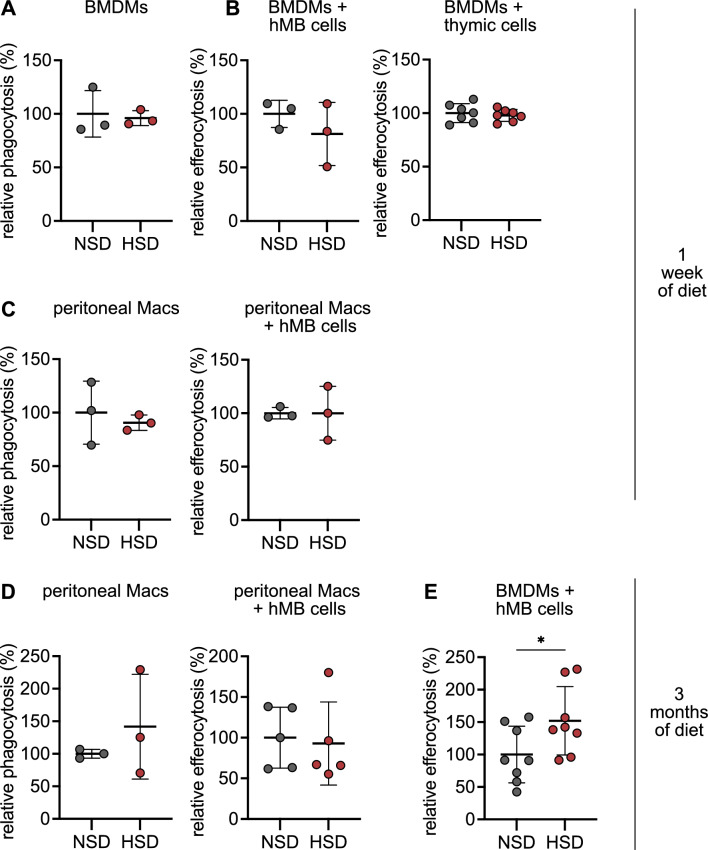
Phagocytosis and efferocytosis activity of macrophages in response to sodium chloride (NaCl) in vivo. C57BL/6 mice were fed a control diet (normal-salt diet NSD) or high-salt diet (HSD) for 1 week (**A–C**) or 3 months (**D and E**). **(A and B)** Phagocytosis of polystyrene beads (**A**) or efferocytosis of hMB and thymic cells (**B**) by bone-marrow derived macrophages (BMDMs) isolated from mice after 1 week of diet. (**C and D**) Phagocytosis of polystyrene beads and efferocytosis of hMB cells by peritoneal macrophages (Macs) isolated from mice after 1 week (**C**) or 3 months of diet (**D**). **(E)** Efferocytosis of hMB cells by BMDMs isolated from mice after 3 months of diet. Significance was tested using unpaired Student’s t-test. *p < 0.05. Each circle represents a biological replicate as the mean of three technical replicates. Shown is the mean ± SD

Finally, we set out to understand whether an in vivo high-salt exposure would alter macrophage functions similar to what we have observed in vitro. To this end, we fed mice with either control diet (CD) or a high-salt diet (HSD) for one week or three months, which would represent acute and chronic exposure, respectively. Using a similar experimental setup as with macrophages treated with NaCl for 24 h, we assessed phagocytosis and efferocytosis of BMDMs and peritoneal macrophages from mice after diet. Intriguingly, there was no difference in phagocytosis (Fig. 4A) or efferocytosis activity using PS beads or apoptotic thymic cells after one week of HSD (Fig. 4B). Only the uptake of hMB cells was slightly decreased in BMDMs (Fig. 4B). We hypothesized that the effect of HSD may be masked by one week of in vitro differentiation of BMDMs, and therefore, assessed the phagocytosis and efferocytosis activity of peritoneal macrophages isolated from mice fed with CD or HSD. Here, we observed no difference in PS uptake or hMB engulfment (Fig. 4C). To address whether the short-term exposure of one week was not sufficient to reprogram macrophages and/or macrophage progenitors, we fed mice with HSD or CD for 3 months. Intriguingly, the chronic exposure to HSD did not impact PS or hMB cell engulfment by peritoneal macrophages, possibly indicating an adaptation process to the high-salt condition (Fig. 4D). However, BMDMs showed an increased efferocytosis of hMB cells (Fig. 4E), indicating that macrophage precursors in the bone marrow were reprogrammed by the HSD conditions. This is in line with recently published work showing that two months of HSD impedes BMDM function but not microglia, the tissue-resident cells of the brain [10]. Intriguingly, sodium concentrations in the bone marrow are controlled by the myeloid cell-derived transcription factor NFAT5, which in turn has a direct impact on osteoclastogenesis [23]. Thus, it remains to be investigated which mechanisms are at play when hematopoietic stem and progenitor cells sense NaCl and how this, in turn, controls differentiation and function of the immune cell compartment in tissues. A previous study has assessed the role of excess NaCl on immune responses indicating that increased osmolarity (100 mM NaCl) activates the inflammasome and, thereby, caspase-1, in macrophages in vitro and that HSD for four weeks promotes a Th17 response in a caspase-1-dependent manner in vivo [24]. It would be interesting to address whether these T cell responses observed in vivo are indeed driven by inflammasome activation in tissue-resident macrophages upon HSD, e.g., using a macrophage-specific knockout of caspase-1.

## Conclusion

In conclusion, our study delves into the impact of dietary salt on macrophage functions, revealing dynamic responses to excess salt both in vitro and in vivo. While acute exposure to high-salt conditions in vitro alters macrophage core functions such as migration, phagocytosis, and efferocytosis, prolonged in vivo salt exposure demonstrates a subtle impact on these functions in BMDMs and peritoneal macrophages. The discrepancies suggest that the sensing and response to salt by macrophages are intricately influenced by the physiological environment. Therefore, comprehensive understanding of the physiological relevance of excess salt on tissue and cell function requires further exploration through in vivo and ex vivo studies, highlighting the imperative nature of these approaches for unravelling the complexities of salt-mediated immune modulation and its implications in disease pathogenesis.

## Data Availability

We have submitted the RNA-seq dataset to GEO database (accession number: GSE250604). Code will be made available by the corresponding authors upon reasonable request.

## Abbreviations

BMDMs: Bone-marrow-derived macrophages
C5a: Complement component 5A
CD: Control diet
DEG: Differentially expressed genes
FcγR: Fc gamma receptors
GO: Gene ontology
HSD: High-salt diet
NaCl: Sodium chloride
PS: Polystyrene
